# Life Insurance Examinations

**Published:** 1906-08

**Authors:** Wilson Williams

**Affiliations:** General Field Superintendent Security Mutual Life Insurance Company, of Binghampton, N. Y.


### LIFE INSURANCE EXAMINATIONS.

By WILSON WILLIAMS,

General Field Superintendent Security Mutual Life Insurance Company, of Binghamton, N. Y.

Ever since the giant life insurance companies reduced examination fees on $1,000 and $2,000 cases, thereby establishing a graded schedule, the county medical societies of some States have been meeting and passing resolutions binding its members to make no examinations for legal reserve (Old Line) companies, requiring urinalysis, for a less fee than $5, irrespective of the amount of policy applied for. In most cases this action, when taken, was done without much deliberation, or even considering but one side of the argument, it being assumed by physicians subscribing to the resolution that the $5 flat fee, if demanded, would be paid. Many physicians, holding commissions as examiners, have wisely refused to endorse such a society action, contending that individual members should be free to decide for themselves what compensation was adequate for the time and service rendered. These argued that it was not fair to their medical confreres to bind all members of the Society in an iron-bound fee schedule for life insurance work or any other, and many, when questioned, have readily acknowledged the fact that the new schedule of fees adopted for medical examinations paid the physician as well (if not better) than any other service performed, not requiring any more time or skill, and considering the further fact of its being sure money.

The Armstrong Investigating Committee revealed many extravagances in company management, which recent enactments will prevent in the future, but the examiner rebels at being affected by a *necessary* reduction in first year's cost of new business. He argues the necessity of an examination, the responsibility which rests upon the examiner, and emphasizes the fact that it requires as much time, care and skill to examine a one-thousand dollar case os it does a five-thousand dollar one. The latter argument, however, fails to take into consideration the fact that of every premium collected from the policy-holder a certain portion is for expense, and on a $5,000 case the company gets five times the expense income that is provided on a one thousand dollar case, and hence can better afford to pay more for the examination. What the company can afford to pay is paid on *all* examinations made, and the average, which should count with the examiner, is more than $3 to the case. Further it must be considered that the examiner's fee is paid, whether the applicant is accepted or rejected, and that on rejected cases the companies receive nothing from the expense incurred.

The cost of examining 100 cases on the graded fee basis is about $333—examinations which provide for a $5 fee placing the average above $3 to the case.

Of the 100 cases examined 10 to 12 will be rejected, and 12 to 15 on which policies are issued will not be placed. This nets the company about 75 per cent. of the total cases examined, out of which to get an expense income sufficient to justify the first year's cost of *all* new business written.

The new insurance laws of the State of New York limit particularly the cost of new business, and prescribe the amount available for all expenses in securing such new business. The companies face a condition and not a theory—a case of "have to" get business within a specified limit of cost, or the executive officer pays the penalty of a misdemeanor.

The average premium per $1,000 insurance is $30 and the available expense for each $1,000 policy written, delivered and paid for is fixed at 66 per cent., or $19.80. With this expense fund, derived only on premiums collected, delivered, business provision must be made for the expense incurred on rejected and not taken cases, as well as on the policy delivered, agents' commission, agency maintenance, and inspections.

Assuming agent's commission and the cost of training new agents in the business to be 50 per cent. of the premium, namely $15, there is left only $4.80 to cover the cost of medical examination and inspection. It is thus apparent that companies could not pay a flat fee of $5 for the examination alone, and pay this irrespective of whether the applicant was accepted or rejected. At the graded fee the $1,000 cases show a deficit of 70 cents, which is about balanced by a gain on the $2,000 cases.

The examiner who argues that his services are necessary to the agent in order to assure his (the agent's) commissions, fails to consider that the agent's entire time is devoted to selling life insurance (or trying to), while examining cases written are but incidents to the physician's practice. Also, that the agent derives no compensation on the cases rejected, while the examiner receives his fee notwithstanding, and that it has probably required days and weeks of persevering endeavor for the agent to get the case up to the examiner, which may then fail to pass. Further, out of the commission made by the agent he has to support his family and pay his own expenses while on the road, while the general practice of the examiner provides amply for his living and office expenses.

Dr. Bross, of the "Equitable," is authority for the statement that 85 per cent. of the total business done in the United States in 1904, the last year before the insurance investigation, was done by companies which have now adopted some sort of graded fee schedule, and only 15 per cent. of the Old Line business by companies now operating under a flat fee. Unless the companies of the 15 per cent. class are holding to the flat fee as capital for trading on the influence of the profession, and this be continued without regard to policyholders' interest, it would appear certain that they would find it necessary in the near future to adopt a graded fee schedule, and the examiner, *if* a *policyholder,* would consider this measure a needed reform of the business.

If physicians of the country should decide to form combinations to compel the payment of a flat fee of $5 on all examinations, those companies which are desirous of doing best by its policyholders will be compelled to unite in the employment of salaried medical examiners, one for each district, or devise some plan for writing insurance under which medical examinations will be done away with. One of the intermediate companies (as regards size) has recently discontinued examinations on $1,000 cases, and how many others will follow will depend largely upon the ability to secure the examiner's services at a cost which the business will warrant.

